# Severe Atypical Conjunctivitis: A Novel Ophthalmic Presentation of Erdheim-Chester Disease

**DOI:** 10.22336/rjo.2026.65

**Published:** 2026

**Authors:** Andrea Córdoba, Javier Andres Díaz-Vargas, Juan Manuel Correa Alvarez, Pedro Jose Ramirez Wiesner, Camilo Restrepo-Perdomo, Mauricio Jaramillo, Miguel Cuevas

**Affiliations:** 1 Cornea Service, Ophthalmology Department, School of Medicine, CES University – CLOFAN Clinic - Medellin, Colombia; 2 Ophthalmology Department, School of Medicine, CES University - Medellin, Colombia; 3 Ophthalmic Pathology Service, Pathology Department, School of Medicine, University of Antioquia - Medellin, Colombia; 4 Oncology Service, Ophthalmology Department, School of Medicine, CES University – CLOFAN Clinic - Medellin, Colombia

**Keywords:** Erdheim-Chester Disease, histiocytosis, non-Langerhans cell histiocytosis, atypical conjunctivitis, ECD = Erdheim-Chester Disease, OS = left eye, OD = right eye, H&E = Hematoxylin-eosin

## Abstract

**Objective:**

To describe an atypical ophthalmological presentation of Erdheim-Chester disease characterized by conjunctival involvement.

**Methods:**

Interventional case report. A 27-year-old woman presented with severe bilateral atypical conjunctivitis associated with a rubbery subconjunctival nodule in the inferior fornix, which led to a histopathological diagnosis of Erdheim-Chester disease.

**Results:**

We performed an excisional biopsy of a subconjunctival nodule. Histological analysis revealed an inflammatory infiltrate with a predominance of large histiocytes with large foamy cytoplasm, CD68+, CD163+, CD1a-, and S100-; confirming Erdheim-Chester disease.

**Discussion:**

Ophthalmological involvement in Erdheim-Chester disease typically manifests as eyelid xanthelasmas and intraconal orbital masses; conjunctival involvement is rarely described. Our patient presented predominantly with severe bilateral refractory conjunctivitis, a yellowish subconjunctival nodule, and secondary corneal epithelial defects. Recognizing atypical conjunctival manifestations facilitates earlier diagnosis of this systemic disease. Multidisciplinary management with systemic polychemotherapy, surgical debridement, and topical 1% 5-fluorouracil achieved marked clinical improvement without relapse after 4 months.

**Conclusion:**

Erdheim-Chester disease is rare and commonly involves the eyes. Although infrequently affected, the conjunctiva may present with subconjunctival nodules or atypical conjunctivitis. Ophthalmologists must remain vigilant for these presentations, as early recognition is crucial for timely diagnosis and appropriate management.

## Introduction

Erdheim-Chester disease (ECD) is a rare condition within the group of non-Langerhans cell histiocytosis, characterized by the infiltration of multiple tissues, organs, and systems by foamy, xanthomatous, CD68-positive, CD1a-/S100-negative histiocytes [1].

Bone involvement is the most common manifestation, occurring in 74 to 92% of cases, and is characterized by multifocal osteosclerotic lesions in the long bones. Other frequently affected areas include the cardiovascular, neurological, and respiratory systems, as well as the retroperitoneal organs and the skin. However, any tissue can potentially be involved [2].

Ophthalmological manifestations are common, occurring in 25 to 30% of cases, and may be the initial presentation. When these manifestations occur, the most frequent are eyelid xanthelasmas and orbital intraconal masses causing exophthalmos and/or ophthalmoplegia [3,4]. Intraocular involvement is rare, but cases of anterior uveitis, vitreitis, optic nerve edema, drusen-like deposits, choroidal infiltration, recurrent serous retinal detachments, and retinal pigment epithelium changes have been reported [2].

To our knowledge, this is the first reported case in which the ophthalmological presentation of ECD was atypical, severe, and different from all previously reported presentations in the literature. In this case, the disease primarily affected the conjunctival and corneal tissues, manifesting as severe atypical conjunctivitis with corneal epithelial defects in both eyes.

## Case report

A 27-year-old female was referred to our cornea service for bilateral, severe, and refractory conjunctivitis of unclear etiology. During the clinical interview, the patient reported a 6-month history of intermittent fever, lymphadenopathy, joint pain, and ulcerated skin plaques on the extremities and the trunk. She had previously been hospitalized and underwent extensive studies with internal medicine, rheumatology, infectious diseases specialists, and dermatology, but a clear diagnosis could not be made.

At the time of the initial assessment, the patient had been experiencing bilateral red eyes for two months, accompanied by abundant conjunctival discharge, ocular pain, and intense photophobia. Despite treatment with multiple topical antibiotics, steroids, and ocular lubricants, her symptoms showed no improvement.

Upon initial examination, corrected visual acuity was 20/60 in the right eye (OD) and 20/25 in the left eye (OS), with preserved eye movements. Biomicroscopy revealed hyperemic conjunctiva with severe follicular reaction, abundant yellowish-white secretion with a creamy, paste-like appearance that was easily removed, and no evidence of membranes or pseudomembranes. Additionally, the OD showed an oval corneal epithelial defect with calcific deposits (**Fig. 1A**). There was no evidence of intraocular inflammation, and no abnormalities were found in the posterior pole. A conjunctival secretion sample was collected for Gram stain, KOH, and cultures, which revealed a moderate leukocyte reaction with no microbial growth.

**Fig. 1:**
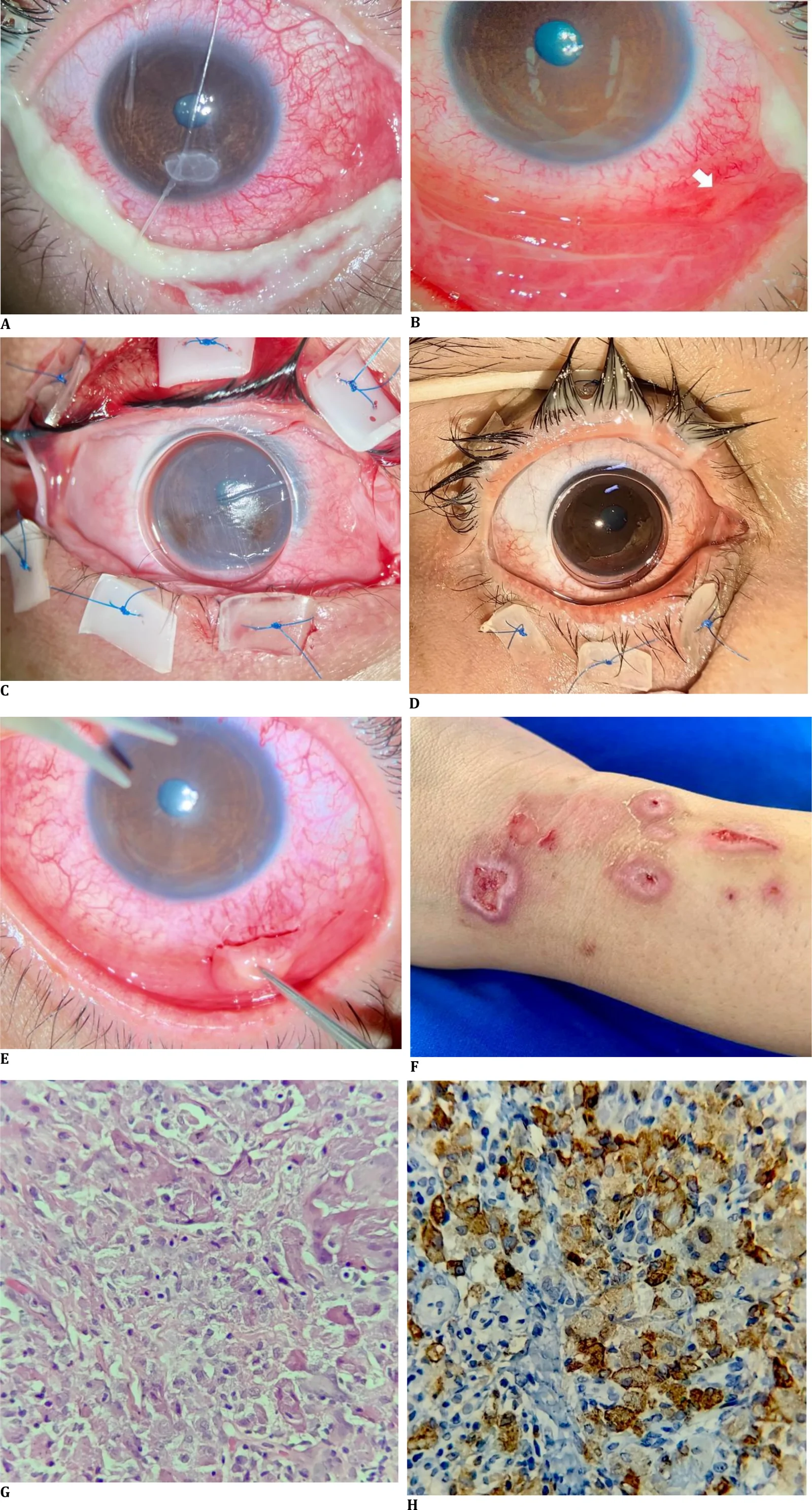
**A** Slit-lamp photography showing the OD with hyperemic conjunctiva, abundant yellowish-white secretion with a creamy, paste-like appearance, and an oval epithelial defect with calcific deposits; **B.** Slit-lamp photography showing the OS with a rubbery and yellow subconjunctival mass in the lower fornix; **C.** Immediate post-surgical appearance of the OD after extensive cleaning of the conjunctival secretion, keratectomy, amniotic membrane graft and symblepharon ring; **D.** 3 weeks post-surgical appearance of the OD after extensive cleaning of the conjunctival secretion, keratectomy, amniotic membrane graft and symblepharon ring; **E.** Intra-surgical appearance of the OS during sub-conjunctival nodule resection; **F.** Ulcerated skin plaques on the hand and forearm; **G.** Histopathological analysis demonstrates a dense mononuclear inflammatory infiltrate, predominantly composed of large histiocytes with foamy cytoplasm, accompanied by plasmocytes, lymphocytes, and multinucleated foreign body-like giant cells. Hematoxylin-eosin (H&E) staining (40X); **H.** Immunohistochemical positivity in histiocytes for CD163 (40X)

The patient was initially treated with preservative-free ocular lubricants every 2 hours and prednisolone 1% three times daily in both eyes, plus topical insulin (4U/ml) five times daily in OD. Weekly follow-up showed no significant changes during the first 2 weeks. After 3 weeks, we observed a rubbery, yellow, ulcerated subconjunctival nodule measuring 2 x 1.5 mm in the lower fornix of the OS (**Fig. 1B**). Given the lack of progress with the established treatment, we decided on surgical intervention. Under general anesthesia, extensive cleaning of conjunctival secretions in both eyes was performed, along with keratectomy to remove calcific material from the epithelial defect in the OD. We placed an amniotic membrane graft on the corneal surface of the OD, fixed it to the edges of the upper and lower eyelids, and secured it with a symblepharon ring (**Fig. 1C, D**).

Additionally, the subconjunctival nodule in the OS was resected for histopathological analysis (**Fig. 1E**). Dermatology was also consulted for a new skin biopsy, as the skin lesions had become active again (**Fig. 1F**).

Histopathological analysis of the subconjunctival nodule revealed a dense inflammatory infiltrate, predominantly composed of large histiocytes with foamy cytoplasm, accompanied by plasmocytes, lymphocytes, and multinucleated foreign body-like giant cells (**Fig. 1G**). Immunohistochemical analysis showed strong positivity in the histiocytes for CD68 and CD163, without S100 or CD1a expression (**Fig. 1H**). This finding was further supported by a skin biopsy, which also identified CD68+, CD163+, and S100- histiocytes in the dermis. This led to a diagnosis of non-Langerhans cell histiocytosis, Erdheim-Chester type. Following this diagnosis, the hemato-oncology department referred the patient for inpatient management.

Multiple extension studies documented bone, lymph node, skin, and ophthalmological involvement. The team ruled out central nervous system, cardiovascular, pulmonary, and renal involvement. Molecular analysis of peripheral blood was negative for BRAF mutations. Hematology initiated treatment with polychemotherapy (Vinblastine/Prednisolone/Cytarabine) for 12 cycles. Additionally, topical chemotherapy with 5-fluorouracil 1% was started for both eyes, with 3 cycles planned.

The ophthalmological outcome after surgery and the initiation of systemic and topical chemotherapy was satisfactory. The OD improved significantly with almost complete remission of secretion after surgery. The OS showed increased secretion during the initial days of hospitalization, along with an epithelial defect with calcific deposits; after 1 month of systemic and topical treatment, the ocular surface improved significantly, secretion resolved completely, and a keratectomy was performed to remove the calcium deposits. A contact lens was left in for one week.

After 4 months of chemotherapy initiation, the patient showed no signs of ophthalmological relapse.

## Discussion

Non-Langerhans cell histiocytosis of the Erdheim-Chester type is an extremely rare but potentially life-threatening condition [1,2]. Ophthalmological involvement is not uncommon, with the most common manifestations being xanthelasmas and intraconal orbital masses, often associated with bilateral and symmetrical exophthalmos, with or without ophthalmoplegia [3,4]. However, because histiocytes can infiltrate any organ or system in this disease, any ocular tissue could be affected.

Other ophthalmological presentations previously described include:

- Corneal and scleral involvement: One case of corneal involvement has been reported, where xanthogranulomatous non-necrotizing inflammation affected the cornea and the sclera, with obliteration of the anterior chamber and angle structures [5]. Another case also reported xanthogranulomatous infiltration of the cornea at the limbus level [6]. Scleral involvement has also been described as scleral nodules and diffuse scleral thickening [7].
- Anterior chamber involvement: Manifests as anterior uveitis [2].
- Vitreous involvement: Presented as vitreitis [2].
- Chorioretinal involvement: Choroidal folds associated with intraconal orbital masses have been described, along with choroidal infiltration by histiocytes, which appear as yellow-white lesions. Recurrent serous retinal detachment may complicate these. Drusen-like deposits of histiocytic material and changes in the retinal pigment epithelium, including exudative material accumulation, pigment deposits, and focal atrophy, have also been observed [2].
- Optic nerve involvement: Optic nerve edema with subsequent atrophy has been reported, usually associated with compressive intraconal masses [2].

Very little has been described about conjunctival involvement in this entity. However, because of its vascularization, the conjunctiva is commonly affected in inflammatory, autoimmune, and infiltrative entities.

Subconjunctival nodules can present in ECD. Although few cases have been specifically reported in ECD [8], similar cases have been described in conditions such as non-Langerhans cell histiocytosis of the Rosai-Dorfman type [9-12] and Langerhans cell histiocytosis [13,14]. These subconjunctival and epibulbar masses typically present in two forms: the first, as erythematous, highly inflammatory, vascularized nodules at the episcleral level, primarily observed in patients with Rosai-Dorfman disease; and the second, as yellowish, less inflammatory, less vascularized nodules with a rubbery consistency. We observed the latter presentation in our patient, which facilitated the histopathological diagnosis of ECD.

The most striking aspect of the ophthalmological presentation in this case was the severe, atypical, bilateral conjunctivitis. This presentation had not been previously described in the literature and was the main reason the patient sought consultation. This conjunctivitis was characterized by a high volume of creamy yellowish secretion that could be gently removed but reappeared within minutes. Based on the prior treatments the patient had received and the management initiated in our service, these findings suggest that this conjunctivitis was highly resistant to steroid therapy.

Significant improvement was observed in the OD following surgery and in the OS after the initiation of systemic and topical chemotherapy.

While the literature describes a case of ECD with conjunctivitis as part of the presentation, that case involved conjunctival inflammation in a patient with bilateral proptosis, facial edema, and mastoiditis—a clinical presentation distinctly different from our patient’s [15]. To our knowledge, no other cases of conjunctivitis as a primary manifestation of ECD have been reported.

In both eyes, possibly due to the chronicity of the condition and the volume and consistency of the secretion, the patient developed epithelial defects in the inferior hemi-corneas. These defects later developed calcific deposits in both eyes and required surgical management.

## Conclusion

Although ECD is rare, ophthalmological involvement is common and diverse. The conjunctiva can be affected, presenting with subconjunctival nodules or atypical conjunctivitis. Ophthalmologists should recognize these presentations, as early recognition is crucial for accurate diagnosis and timely ECD treatment.
